# An in vitro screening cascade to identify neuroprotective antioxidants in ALS

**DOI:** 10.1016/j.freeradbiomed.2009.01.019

**Published:** 2009-04-15

**Authors:** Siân C. Barber, Adrian Higginbottom, Richard J. Mead, Stuart Barber, Pamela J. Shaw

**Affiliations:** a Academic Neurology Unit and Sheffield Care and Research Centre for Motor Neuron Disorders, University of Sheffield, Sheffield S10 2RX, UK; b Department of Statistics, University of Leeds, Leeds, UK

**Keywords:** 5-LOX, 5-lipoxygenase, AAPH, 2,2′-azobis(2-methylpropionamidine) dihydrochloride, ALS, amyotrophic lateral sclerosis, ARE, antioxidant response element, BBB, blood–brain barrier, CAPE, caffeic acid phenethyl ester, CNS, central nervous system, DCF, dichlorofluorescein, DMSO, dimethyl sulfoxide, Esc, esculetin, EthD1, ethidium homodimer-1, EGFP, enhanced green fluorescent protein, LTB_4_, leukotriene B_4_, MN, motor neuron, MTT, methylthiazolyldiphenyl tetrazolium bromide, NDGA, nordihydroguaiaretic acid, Nrf2, nuclear factor erythroid 2-related factor 2, OTCA, 2-oxo-l-thiazolidine-4-carboxylic acid, PBS, phosphate-buffered saline, PI, prediction interval, PSA, polar surface area, Res, resveratrol, R-PE, R-phycoerythrin, SOD1, superoxide dismutase 1, TK, thymidine kinase promoter, TRAP, total radical-trapping antioxidant parameter., Antioxidant, Amyotrophic lateral sclerosis, Mouse, NSC34, Superoxide dismutase, Free radicals

## Abstract

Amyotrophic lateral sclerosis (ALS) is an adult-onset neurodegenerative disease, characterized by progressive dysfunction and death of motor neurons. Although evidence for oxidative stress in ALS pathogenesis is well described, antioxidants have generally shown poor efficacy in animal models and human clinical trials. We have developed an in vitro screening cascade to identify antioxidant molecules capable of rescuing NSC34 motor neuron cells expressing an ALS-associated mutation of superoxide dismutase 1. We have tested known antioxidants and screened a library of 2000 small molecules. The library screen identified 164 antioxidant molecules, which were refined to the 9 most promising molecules in subsequent experiments. Analysis of the in silico properties of hit compounds and a review of published literature on their in vivo effectiveness have enabled us to systematically identify molecules with antioxidant activity combined with chemical properties necessary to penetrate the central nervous system. The top-performing molecules identified include caffeic acid phenethyl ester, esculetin, and resveratrol. These compounds were tested for their ability to rescue primary motor neuron cultures after trophic factor withdrawal, and the mechanisms of action of their antioxidant effects were investigated. Subsequent in vivo studies can be targeted using molecules with the greatest probability of success.

Amyotrophic lateral sclerosis (ALS)^1^ is an adult-onset neurodegenerative disease characterized by progressive dysfunction and death of motor neurons (MNs) in the motor cortex, brain stem, and spinal cord. The cause of disease is unknown in the majority of cases, classified as sporadic ALS, but approximately 10% of cases have a genetic cause. ALS-causing mutations have been identified in several genes, of which the best described is mutation of Cu,Zn superoxide dismutase (SOD1), responsible for approximately 20% of familial cases [1]. Sporadic and familial ALS are clinically indistinguishable and there is good evidence to suggest that they share a common pathogenic mechanism that includes oxidative stress, excitotoxicity, mitochondrial dysfunction, protein aggregation, axonal transport defects, and inflammatory cascades [2]. Precisely how mutations in SOD1 lead to MN death is still unclear, although there are likely to be multiple mechanisms because over 100 disease-causing mutations have been identified in the 154-amino-acid protein. Some SOD1 mutants develop a free radical-generating ability (reviewed in [3]), and mutant SOD1 has recently been shown to cause sustained activation of NADPH oxidase, leading to increased superoxide production [4], and to induce endoplasmic reticulum stress, leading to apoptosis [5,6]. Increasing evidence has indicated that non-cell-autonomous mechanisms are also involved, requiring the presence of mutant SOD1 in neurons and glia [7–9], although a recent study showed that neuronal expression of mutant SOD1 is sufficient to induce an ALS phenotype in transgenic mice [10].

The involvement of oxidative stress in the pathogenesis of ALS has been described extensively, with oxidative damage to DNA, proteins, and lipids within pathologically affected areas of the central nervous system (CNS) [11–13], which is reproduced in animal and cell culture models [14–16]. Although injurious in its own right, oxidative stress can also exacerbate other mechanisms contributing to neurodegeneration in ALS [3,17–20]. Therefore, whether oxidative stress is a primary cause of neurodegeneration, or merely a secondary consequence of another toxic insult, the evidence suggests that it is a major contributory factor leading to MN loss in ALS, making oxidative stress an attractive therapeutic target. However, antioxidants have not performed well in clinical trials [21,22], leading to the suggestion that they may not be effective in ALS. A closer examination of published reports suggests that may not be the case. The mutant SOD1 mouse model [23] has long been considered the “gold standard” for preclinical drug testing in ALS. However, 14 years after its development, and with over 150 drugs tested, no treatment has successfully translated to the clinic, raising doubts about the rigor of the drug trials and the relevance of the mouse model to human ALS [24–26]. Given that, a recent meta-analysis of drug treatment trials in the mutant SOD1 mouse model of ALS concluded that antioxidant drugs have been the most effective drug group in terms of extending survival among animals treated at symptom onset [24]. The poor success of antioxidants in clinical trials may also be partly due to failure to achieve drug concentrations in the CNS sufficient to have a therapeutic effect. For example, trials of vitamin E, which does not readily penetrate the CNS [27], have failed to show a robust beneficial effect in ALS even when administered at high dose [28].

If an antioxidant therapy is to progress beyond experimental systems and move into the clinic, it is necessary to consider not only the antioxidant capacity of the molecule, but also its physical and chemical properties to obtain a concentration at the target cell population sufficient to achieve a worthwhile neuroprotective effect. We have developed a systematic in vitro screening strategy for identifying effective antioxidant molecules predicted to have the ability to access the CNS, which may have a therapeutic benefit for ALS patients. Molecules that showed good efficacy in the in vitro systems were then screened in silico for their potential ability to successfully enter the CNS, and the mechanism(s) by which they act were investigated further. Three molecules, caffeic acid phenethyl ester (CAPE), esculetin, and resveratrol, were identified as effective antioxidants with the ability to increase the viability of a mutant SOD1-expressing cell line and with biochemical properties indicating they can penetrate the CNS.

## Materials and methods

General laboratory reagents were from Sigma. Tissue culture reagents were from Invitrogen, except fetal bovine serum (FBS), from BioSera (Ringmer, East Sussex, UK). Tissue culture flasks and plates were from Greiner (Stonehouse, Gloucestershire, UK).

### Cell culture

NSC34 mouse motor neuron cells [29] were maintained in DMEM containing 10% fetal bovine serum (D10). NSC34 cells were transfected with pIRESneo (Clontech, Saint-Germain, France) using Lipofectamine 2000 (Invitrogen). Cells were transfected with empty vector (pIRES cells) or pIRESneo containing wild-type human SOD1 (SOD1 cells) or one of four human SOD1 mutants (G37R, H48Q, G93A, and I113T cells). G418 (250 μg/ml) was added to cells 24 h later to select for transfected cells. Single cell clones expressing comparable levels of human SOD1, determined by Western blotting using sheep anti-mouse SOD1 polyclonal antibody (Calbiochem, Nottingham, UK), were selected.

### Oxidative stress assay

NSC34 cells were grown in 96- or 384-well tissue-culture plates in phenol red-free D10 until 30% confluent. Oxidative stress was induced by 3 h serum withdrawal, in the presence or absence of compound. Cytosolic reactive oxygen species levels were measured using dichlorofluorescein (DCF) fluorescence. Carboxy-H_2_DCFDA (6-carboxy-2′,7′-dichlorodihydrofluorescein diacetate, di(acetoxymethyl ester); Molecular Probes, Paisley, UK) was added to NSC34 cells to 5 μM, and the fluorescence of oxidized DCF was read at Ex_485 nm_/Em_530 nm_ after 1 h using a Fusion universal plate reader (Packard BioScience, Beaconsfield, Buckinghamshire, UK). Cell death was simultaneously measured by adding ethidium homodimer-1 (EthD1; 0.3 μM; Molecular Probes) to the culture medium, and fluorescence was measured at Ex_530 nm_/Em_645 nm_. When different cell lines were compared, DCF results were normalized to cell number, determined by measuring EthD1 fluorescence after cells had been freeze–thawed. The Spectrum Collection (Microsource Discovery Systems, Gaylordsville, CT, USA) was screened using NSC34 cells grown in black-walled 384-well plates using a Q-bot liquid handling robot (Genetix, New Milton, Hampshire, UK). The Spectrum Collection was stored in dimethyl sulfoxide (DMSO) at a concentration of 5 mM. For experiments, DMSO stocks were diluted directly in culture medium and all experiments included a DMSO negative control containing equivalent concentrations of DMSO.

### Cell viability assay

NSC34 cells were grown in 96-well plates in D10 until 60% confluent. Medium was replaced with fresh medium containing 10 μM menadione, to induce oxidative stress [30], and Spectrum library compound for 4 h. Cell viability was assessed using the MTT assay, as described previously [31].

### In silico screening

In silico screening was performed using SciTegic Pipeline Pilot software (Accelrys, Cambridge, UK). The molecular polar surface area (PSA) was calculated for all 2000 molecules from the Spectrum Collection as a crude measure of likely CNS penetrance [32] and a Lipinski filter was also applied to determine which molecules were most drug-like. This filter applies the “Rule of 5” [33], which selects molecules with a log*P* < 5, molecular mass < 500, < 5 hydrogen bond donors (OH + NH count), and < 10 hydrogen bond acceptors (O + N atoms).

### Primary MN cultures

Primary MN cultures were established from E13.5 C57BL/6J mouse embryos using established methods [34]. MN enrichment was performed by layering the cell suspension onto a 6.4% iodixanol cushion (Axis-Shield, Kimbolton, Cambridgeshire, UK) and centrifuging at 500 g for 15 min. The MN-enriched fraction was plated onto poly-d-ornithine/laminin-coated glass chamberslides at 12,000 cells/cm^2^ in MN medium (neurobasal medium containing B27 supplement, 2% horse serum, 1 ng/ml BDNF, 1 ng/ml GDNF, 5 ng/ml CNTF (growth factors from R and D Systems, Abingdon, Oxon, UK)). After 24 h, cultures were washed in phosphate-buffered saline (PBS) and then incubated in MN medium without growth factors or antioxidants, but supplemented with 10 μM compound (or 0.2% DMSO as a vehicle control). After a further 24 h, cultures were washed in PBS and fixed in 4% paraformaldehyde in PBS. Immunocytochemistry was performed on cultures permeabilized in 0.1% Triton X-100 using SMI-31 (Sternberger Monoclonals, Baltimore, MD, USA) and rabbit anti-peripherin (Chemicon International, Watford, Hertfordshire, UK) primary antibodies and donkey anti-rabbit–Alexa Fluor 555 and donkey anti-mouse–Alexa Fluor 488 (Invitrogen) secondary antibodies. Nuclei were labeled using Hoechst 33342. The number of MNs present in 20 random fields was counted for each culture using a 63× oil emersion objective on a fluorescence microscope.

### Total radical-trapping antioxidant parameter (TRAP) assay

Radical-trapping antioxidant activity of compounds was tested using a modification to published methods [35]. The reaction mixture consisted of 5 μM compound and 15 nM R-phycoerythrin (R-PE) in 75 mM phosphate buffer (pH 7.2). The oxidation reaction was started by addition of 2,2′-azobis(2-methylpropionamidine) dihydrochloride (AAPH) to a final concentration of 50 mM. Decay of R-PE fluorescence was measured at Ex_490 nm_/Em_580 nm_ 50 times, with a 1-min delay between reads. Each compound was tested in eight wells and a control without AAPH was also run to control for non-AAPH-mediated R-PE decay. The average fluorescence trace for the no-AAPH control was subtracted from the average trace for each molecule tested to produce a relative fluorescence trace. The lag time for each molecule was defined as the last read at which the fluorescence was equal to or above the lower bound of a 95% confidence interval for the mean starting relative fluorescence.

### Antioxidant response element (ARE) reporter assay

Chinese hamster ovary cells stably expressing 4×ARE-TK-EGFP or TK-EGFP reporter constructs (constructs kindly provided by William E. Fahl, University of Wisconsin [36]) were produced as described elsewhere [37] and were maintained in D10. Confluent cultures of 4×ARE-TK-EGFP- or TK-EGFP-expressing cells in 96-well tissue-culture plates were treated with compound (0.01–100 μM) or vehicle (DMSO) in FBS-free DMEM in triplicate for 24 h. EGFP fluorescence was then measured at Ex_485 nm_/Em_530 nm_.

### 5-Lipoxygenase activity assay

NSC34 cells were grown to 90% confluency in a 24-well plate, washed with PBS, and then incubated in 300 μl/well of DMEM containing N2 supplement, 4 mM Hepes, 10 μM arachidonic acid, and 10 μM compound (or 0.2% DMSO as a control) for 3 h. Conditioned medium was harvested, centrifuged to remove any cellular debris, diluted 1:5 in DMEM, and assayed in triplicate for leukotriene B_4_ (LTB_4_) using an LTB_4_ ELISA (IDS Ltd., Boldon, Tyne and Wear, UK), according to the manufacturer's instructions. Absorbance was measured at 405 nm.

### NF-κB assay

NSC34 cells were grown to 90% confluency and treated with 10 μM compound for 1 h before addition of 10 μM menadione for 5 min. Cells were harvested in ice-cold PBS and nuclear lysates were prepared using the NucBuster protein extraction kit (Novagen, Nottingham, UK). Nuclear extracts (6 μg) were analyzed in triplicate for NF-κB translocation using the NoShift II NF-κB transcription factor assay (Novagen), according to the manufacturer's instructions.

### Statistical analysis

Statistical analysis was performed using R (http://www.R-project.org). In the initial library screen, “hits” were defined as molecules giving a DCF reading below the lower bound of a 99% prediction interval (PI) for observations on the negative control. A 99% PI means that if the experiment was repeated, 99% of negative controls would be expected to fall within the prediction interval. The nonstandard significance level of 99% was used to make the screen more stringent. Fluorescence readings from “blank” wells, containing medium and carboxy-H_2_DCFDA, were subtracted from the raw data before statistical analysis was performed. Further screening was done by excluding toxic molecules, defined as molecules giving an EthD1 reading above the upper bound of a 95% PI for observations on the negative control (after subtraction of fluorescence from blank wells containing medium and EthD1, but no cells). To estimate EC_50_ values, logistic curves were fitted to the data and the relevant concentration values were determined from the fitted curves. If a molecule was toxic at high concentration (based on EthD1 result), those concentrations were removed before curve fitting.

In the G93A-SOD1 assays, the data were checked for normality and equal variance using the Shapiro–Wilk test of normality and Fligner–Killeen test of equal variance. Where normality and equal variance criteria were satisfied, the data were analyzed using ANOVA. For any data for which the ANOVA assumptions were not satisfied, a nonparametric test (Kruskal–Wallis) was used. In either case, hits were defined as molecules that significantly reduced DCF fluorescence (*p* < 0.05).

For all other statistical analyses, the data were tested for normality and equal variance as above. ANOVA was used when the data were normally distributed, and if significance was observed, pairwise Student's *t* tests adjusted for multiple comparisons using the false discovery rate modifier [38] were used to determine which conditions were significantly different from the control. Otherwise, equivalent nonparametric tests (Kruskal–Wallis test followed by pairwise Wilcoxon tests adjusted for multiple comparisons) were used. All pairwise comparisons were carried out using two-tailed statistical tests. Statistical significance was always considered as *p* < 0.05.

## Results

### Development of a motor neuron cell culture model of mSOD1-mediated ALS

**Fig. 1:**
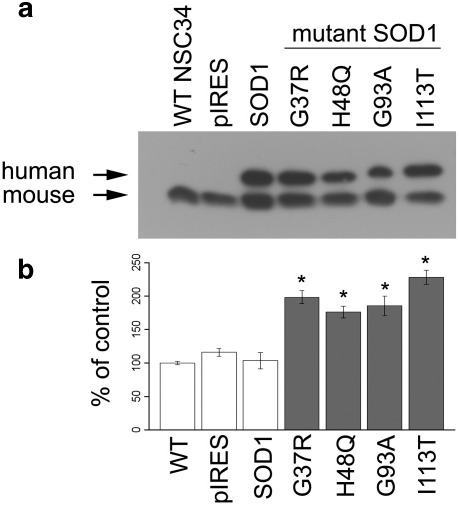
Mutant SOD1 induces oxidative stress in a motor neuron cell culture model. (a) Western blot of NSC34 cell lysates, showing human SOD1 (upper band) and endogenous murine SOD1 (lower band). (b) Basal oxidative stress levels measured by quantification of DCF fluorescence in NSC34 cell lines expressing normal and mutant human SOD1, normalized to cell number. Data shown are means ± SEM. ANOVA discovered significant differences between treatments (*p* = 1.3 × 10^− 7^). There was no significant difference between any of the control cell lines (white bars), but all mutant SOD1-expressing cells (gray bars) have higher intracellular ROS than control cells (*p* < 0.0001, Student's *t* test adjusted for false discovery rate).

An in vitro model of mSOD1-mediated ALS was established by generating single cell clones of NSC34 motor neuron cells stably expressing equivalent amounts of either normal human SOD1 or one of four human SOD1 mutants associated with ALS (Fig. 1a). The SOD1 mutants used represent different classes of mutation: G37R retains full dismutase activity but has reduced polypeptide stability [39]; H48Q has aberrant copper binding and little/no dismutase activity, but does have a superoxide-dependent peroxidase activity, which produces a powerful oxidant [40]; G93A has normal dismutase activity but has increased free radical-generating function [41]; and I113T has reduced dismutase activity [39] and decreased stability due to changes at the dimer interface [42]. Because the precise mechanism(s) by which mutant SOD1 triggers increased oxidative stress is as yet unknown, we measured total oxidative stress by DCF fluorescence. Under basal culture conditions when oxidative stress levels were low, expression of wild-type human SOD1 did not reduce oxidative stress levels relative to untransfected or vector-transfected cells (Fig. 1b), probably because SOD1 enzyme activity was not operating at its maximum rate, so further increasing levels of wild-type SOD1 protein would not alter the oxidative stress balance. However, cytosolic oxidative stress was significantly increased in NSC34 cells expressing any one of the four human SOD1 mutants compared to either wild-type NSC34 cells or cells expressing pIRES vector or normal human SOD1. Given that the SOD1 mutations used here all resulted in increased levels of oxidative stress, this suggests that the increase was not due to novel catalytic activity, but rather due to an unknown toxic gain of function common to all mutants used. These results show that this cell model reproduces the oxidative stress observed in other ALS models and the human disease.

### Simplified assay system for multiple testing

**Fig. 2:**
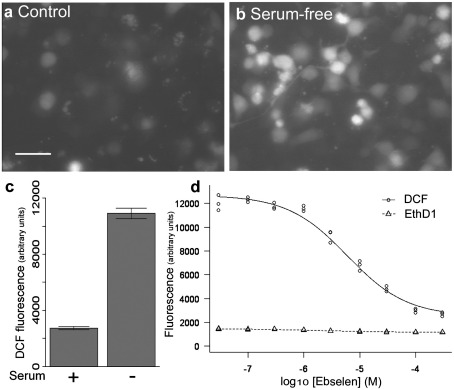
Oxidative stress can be induced in NSC34 cells by serum withdrawal. (a) DCF fluorescence in wild-type NSC34 cells under basal conditions and (b) after serum withdrawal. Scale bar, 50 μm. (c) Serum withdrawal induced a threefold increase in DCF fluorescence in wild-type NSC34 cells. Graph shows means ± 1 SEM. (d) Ebselen reduced serum withdrawal-induced oxidative stress, measured by DCF fluorescence, in NSC34 cells in a dose-dependent manner, with a half-maximal effect (EC_50_) of 4 μM. There was no increase in toxicity, as measured by EthD1 fluorescence.

To efficiently screen large numbers of potential antioxidant compounds, it was necessary to develop a simple cell-based oxidative stress assay with a large window of effect. Short-term serum withdrawal triggers an increase in oxidative stress in wild-type NSC34 cells [43], which was seen as a threefold increase in DCF fluorescence (Figs. 2a–c). This increase in oxidative stress could be rescued by ebselen (Fig. 2d), an organo-selenium antioxidant compound previously shown to be protective against serum withdrawal-induced death in mSOD1-expressing NSC34 cells [37]. Because a reduction in DCF fluorescence would also occur if ebselen induced cell death through a mechanism not involving oxidative stress, a simultaneous toxicity assay was also incorporated. EthD1 fluoresces when bound to DNA, but is not cell permeable and therefore fluoresces only when bound to DNA in a cell with a compromised membrane. Ebselen reduced oxidative stress measured by DCF fluorescence without increasing EthD1 fluorescence, indicating that the reduced DCF fluorescence was caused by a reduction in oxidative stress (Fig. 2d). This simple assay system could then be used to screen larger numbers of potential antioxidant molecules.

### Screening of known antioxidant molecules

Before expanding the assay to screen a compound library, a targeted approach was adopted in which the literature was reviewed for molecules, not present within the Spectrum Collection, that are known to activate a variety of cellular antioxidant mechanisms. These molecules were tested for their ability to reduce serum-withdrawal-induced oxidative stress in NSC34 cells (Table 1). Each molecule was tested at concentrations between 0.03 and 100 μM. The free radical scavenger Trolox (a vitamin E analogue) was effective, whereas other scavengers including the glutathione precursor 2-oxo-l-thiazolidine-4-carboxylic acid (OTCA) and 4-hydroxy-2,2,6,6-tetramethylpiperidine 1-oxyl (Tempol) failed to reduce serum withdrawal-induced oxidative stress. Glutathione, vitamin C, and vitamin E were not tested because they are all present within the Spectrum Collection. Another free radical scavenger, α-lipoic acid, and the NADPH oxidase inhibitor apocynin were also ineffective. The failure of apocynin was not unexpected, because it is thought to act in glial cells rather than neurons [4]. Other molecules found to be capable of reducing oxidative stress in serum-withdrawn NSC34 cells include: flupirtine, which decreases mitochondrial free radical generation; the 5-lipoxygenase antagonist nordihydroguaiaretic acid (NDGA); and the anandamide transport inhibitor AM404. All of the molecules that showed an antioxidant effect in serum-withdrawn NSC34 cells had EC_50_ values between 1 and 10 μM and were capable of reducing the oxidative stress by over 50% (Table 1).

Having identified molecules capable of reducing oxidative stress in the simple cell assay, we then tested these molecules in the more disease-relevant G93A-SOD1-expressing NSC34 cells to determine whether they were effective against the oxidative stress induced by the presence of mutant SOD1. Initially, antioxidant ability was tested using DCF and EthD1 fluorescence. Because the effective window was smaller in this assay system, and only molecules that showed an effect at low micromolar concentrations were of interest, G93A-SOD1-expressing cells were treated with concentrations of each molecule up to 10 μM under basal conditions for 4–6 h and DCF fluorescence was measured as before. Flupirtine was unable to reduce oxidative stress caused by G93A-SOD1, whereas Trolox, NDGA, and AM404 were still effective (Table 1).

To validate these results in a different assay system that did not involve DCF fluorescence, the three molecules that were effective in reducing G93A-SOD1-mediated oxidative stress were then tested for their ability to increase viability of G93A-SOD1-expressing NSC34 cells exposed to a further oxidative stress. This additional oxidative stress was induced using the quinone-containing compound menadione, which undergoes one-electron reduction to produce semiquinone radicals, which react with molecular oxygen to generate reactive oxygen species [30]. After menadione treatment, cell viability was assessed by MTT assay. Treatment with 10 μM menadione for 4 h reduced viability to 10–20% of controls, and this could be rescued only by NDGA and AM404 (Table 1).

### Screening of Spectrum Collection in NSC34 cells

**Fig. 3:**
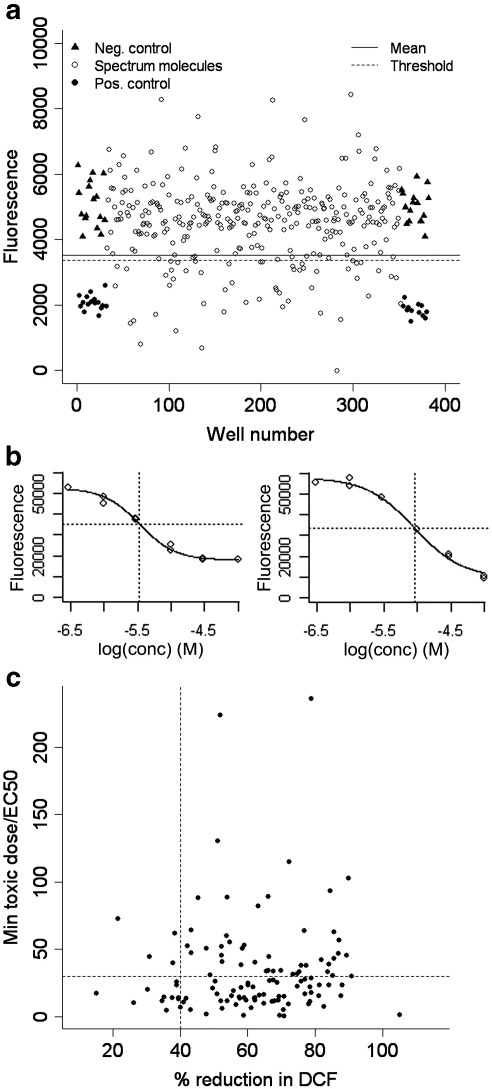
Summary of screening results in NSC34 cells. (a) Results from a typical 384-well plate of NSC34 cells used to screen the Spectrum Collection. The solid line shows the midpoint (mean) between the negative (vehicle, 0.2% DMSO) and the positive control (10 μM ebselen), and the broken line shows the threshold of the lower bound of a 99% prediction interval for observations on the negative control. All molecules giving a DCF fluorescence value below this broken line were classified as hits (provided no toxicity was observed by EthD1 fluorescence). (b) Representative dose–response curves from two molecules. Dotted lines show the concentration giving the half-maximal effect (EC_50_). (c) Plotting % reduction in DCF versus minimum toxic dose/EC_50_ shows which molecules are effective antioxidants with a large window between an effective dose and a toxic dose. Cutoff points of below a 40% reduction in DCF fluorescence and below a toxicity/EC_50_ of 30 produced 45 hits, shown in the top-right quadrant.

Having developed an in vitro screening cascade capable of identifying molecules that reduce oxidative stress and increase viability of motor neuron cells expressing an ALS-associated mutant of SOD1, we used the methodology to screen the Spectrum Collection of 2000 molecules. This collection consists of the National Institute of Neurological Disorders and Stroke (NINDS) Custom Collection, pure natural products, and a small number of endocrine disruptors and toxic substances. The initial library screen was performed using the simple serum-withdrawal oxidative stress in wild-type NSC34 cells. Each molecule was tested at 10 μM on two separate occasions. Vehicle (0.2% DMSO) and 10 μM ebselen were used as negative and positive controls, respectively. Hits were defined as molecules that gave a DCF fluorescence of less than the lower bound of a 99% PI for observations on the negative control. Using this threshold, over 98% of positive controls were successfully identified as hits. A molecule was defined as toxic if it gave an EthD1 fluorescence of greater than the upper bound of a 95% PI for observations on the negative control. Using these criteria, 164 molecules were identified as hits in both runs of the library. The results from a typical 384-well plate are shown in Fig. 3a.

The 164 molecules (and DMSO vehicle) were next tested for their ability to reduce DCF fluorescence in serum-withdrawn NSC34 cells at five concentrations (1, 3, 10, 30, and 100 μM) in duplicate. Toxicity was again tested by measuring EthD1 fluorescence. A result of toxicity was returned for any concentration at which both wells gave an EthD1 fluorescence greater than the upper bound of a 95% PI for observations on the negative control. The DMSO vehicle control was not toxic even at the highest concentration tested (2%). The DCF fluorescence was plotted for each molecule at all concentrations for which toxicity was not observed. No dose response was seen for 42 molecules (and DMSO). For the remaining 122 molecules, the maximum percentage decrease in DCF fluorescence was calculated, and the concentration that produced a 50% effect (EC_50_) was estimated by interpolation using a fitted logistic curve. Representative dose–response curves are shown in Fig. 3b.

Molecules with the potential to be used as antioxidants in vivo must be capable of reducing oxidative stress at low concentration without causing toxicity. To identify such molecules, the data were analyzed in two ways and the molecules were ranked according to two different parameters: (1) antioxidant ability (measured by DCF reduction), regardless of concentration, and (2) index of efficacy relative to toxicity, which considered the window between an effective dose and a toxic dose. This index was produced by dividing the lowest concentration that showed toxicity (for molecules for which toxicity was not seen at the highest concentration tested, toxicity was assumed to be present at the next concentration on the dose curve) by the estimated EC_50_ value. A high value would correspond to a large window between the EC_50_ and toxicity. Plotting the antioxidant ability against the index of efficacy relative to toxicity provided a way to identify the molecules that scored well on both measures. Forty-five molecules were identified as hits that gave a DCF decrease of at least 40% and an index of efficacy to toxicity of over 30 (Fig. 3c). Six further molecules were also selected because they gave estimated EC_50_ values of below 5 μM, and it was considered possible that these molecules could be effective at very low concentrations. These 51 compounds were retested over a more comprehensive concentration range between 0.01 and 100 μM in triplicate. Clear dose–response curves with EC_50_ values below 10 μM were produced by 47 molecules. All molecules that failed showed an effect only at 30 or 100 μM, which was considered to be too high to be of potential therapeutic benefit.

### Validation of hits in G93A-SOD1-expressing cells

The remaining 47 molecules were then tested in NSC34 cells expressing G93A-SOD1, which showed increased oxidative stress under basal conditions. The cells were treated with three concentrations of each molecule (0.1, 1, and 10 μM) under basal conditions for 4–6 h and DCF and EthD1 fluorescence was measured as before. Twenty molecules significantly reduced basal DCF fluorescence in a nontoxic manner in G93A-SOD1 NSC34 cells. Of these, 8 reduced DCF fluorescence in G93A-expressing cells to less than 50% of basal DCF fluorescence at a concentration of 10 μM.

**Fig. 4:**
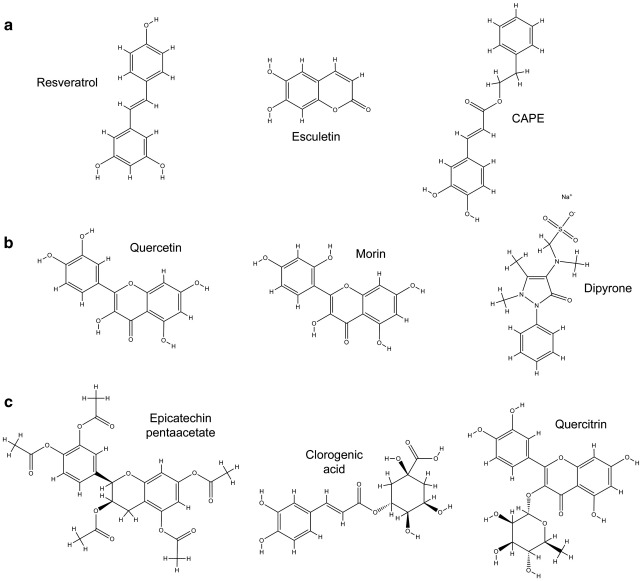
Chemical structures of Spectrum library compounds that had a significant effect in all four in vitro screens and can be tolerated in vivo. Compounds are subdivided according to their predicted biochemical properties (Table 2). (a) Compounds satisfying Lipinski's Rule of 5 for an orally available drug and with a polar surface area (PSA) of < 70 Å^2^. Compounds (b) with a high PSA or (c) that failed the Rule of 5 were not taken further.

The 20 molecules that emerged from the staged screening described above were then tested for their ability to increase viability of G93A-SOD1-expressing NSC34 cells exposed to a further oxidative stress. Treatment with 10 μM menadione for 4 h reduced viability to 10–20% of controls, and this could be significantly rescued by 14 of the 20 molecules tested. Therefore, these molecules not only were capable of reducing oxidative stress, but also could increase viability in oxidatively stressed cells. Of these, 3 doubled the viability of menadione-treated G93A cells, and another 6 increased viability by at least 50%. Three of these top-performing molecules were also in the top-performing molecules in the basal oxidative stress assay. Ebselen, which is represented within the Spectrum Collection, was one of the 6 compounds that was effective in the DCF assays, but failed to significantly increase the survival of menadione-treated NSC34 cells. A review of the literature showed that 9 of the 14 effective compounds have been found to be tolerated in mammalian in vivo systems. These 9 compounds, shown in Fig. 4, formed the hits from the in vitro screening of the Spectrum library. The results of each of these molecules for each stage of the screening process are summarized in Table 2.

### In silico analysis

The biochemical structures of the nine hits were analyzed using SciTegic Pipeline Pilot software to identify the molecules with “drug-like” properties (Table 2). Six molecules passed the Rule of 5, which states that most drug-like molecules have a molecular weight of 500 or less, a log*P* value (octanol/water partition coefficient; a measure of molecular hydrophobicity) of 5 or less, up to 5 hydrogen bond donors, and up to 10 hydrogen bond acceptors [33]. The molecular PSA of a molecule is a predictor for blood–brain barrier (BBB) penetration [32], with the majority of orally administered CNS-active drugs that penetrate the BBB passively having a PSA of below 70 Å^2^ and virtually all having a PSA below 120 Å^2^ [44]. Three of the molecules that passed the Rule of 5 had a PSA of less than 70 Å^2^.

**Fig. 5:**
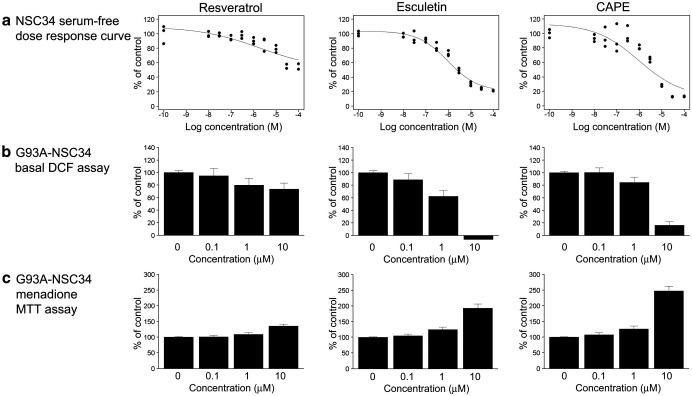
Summary of the performances of the top molecules in the in vitro screening experiments. (a) NSC34 serum-free dose–response curves. (b) G93A-SOD1 NSC34 basal DCF assay results (mean + SEM). Where a negative result is shown, the DCF fluorescence was reduced to levels below background fluorescence levels in blank wells containing medium and carboxy-H_2_DCFDA, but no cells. (c) G93A-SOD1 NSC34 menadione MTT viability assay results (mean + SEM).

The three compounds that passed these criteria were resveratrol, esculetin, and CAPE (Fig. 4a). All three molecules have previously been identified as having antioxidant activity [45–50]. Of the six remaining molecules, three passed the Rule of 5 but had PSA values above 70 Å^2^ (Fig. 4b) and three failed both the Rule of 5 and the PSA criteria (Fig. 4c), making them less likely to be effective in vivo, and were therefore not studied further. In silico analysis of the molecules identified in the screen of known antioxidants showed that NDGA passed the Rule of 5, but had a molecular PSA of 80.9 Å^2^, whereas AM404 failed the Rule of 5. Because neither of these molecules passed the in silico criteria, they were not taken forward for further study. The compounds taken forward from the in vitro screen of the Spectrum Collection were resveratrol, esculetin, and CAPE. Typical results for each of these molecules from each stage of the screening process are shown in Fig. 5.

### Primary MN assay

**Fig. 6:**
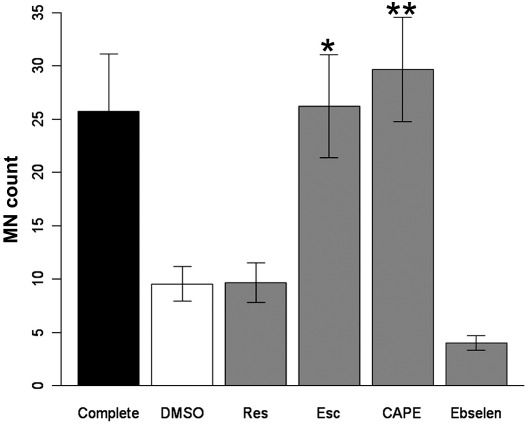
Effects of the best-hit molecules on survival of primary mouse motor neurons deprived of growth factors. Results show the mean numbers of motor neurons ± SEM counted in 20 random fields from four cultures. All conditions used medium without growth factors or antioxidants, except “Complete,” which contained complete motor neuron medium, as defined under Materials and methods. ⁎*p* = 0.020, ⁎⁎*p* = 0.014 (Wilcoxon test, adjusted for false discovery rate, after significant results from a Kruskal–Wallis test (*p* = 0.0005)).

Having identified molecules capable of rescuing oxidative stress associated with G93A-SOD1 in a motor neuron cell line, we then tested the molecules for their ability to support survival of primary MN cultures in which oxidative stress had been induced by trophic factor withdrawal [51]. Primary MNs were cultured from mouse embryos for 24 h, after which trophic factors and antioxidants were withdrawn for a further 24 h. Cultures deprived of trophic factors and antioxidants showed significantly decreased viability, as assessed by counting the number of surviving MNs in 20 random fields (42.75 ± 6.25% of control cultures (mean ± SEM), *p* = 0.007, Wilcoxon test). Addition of either CAPE or esculetin at 10 μM significantly increased MN viability (*p* = 0.014 and 0.020, respectively; Fig. 6). Neither resveratrol nor ebselen was capable of increasing MN viability.

### Mechanism of action of best-hit molecules

Because the best-hit molecules were identified through assays measuring total oxidative stress and viability, it was not known how they were acting. To investigate whether the best-hit molecules act through a common mechanism or activate diverse pathways the effect of each compound was tested on several classical antioxidant pathways.

#### Free radical buffering

**Fig. 7:**
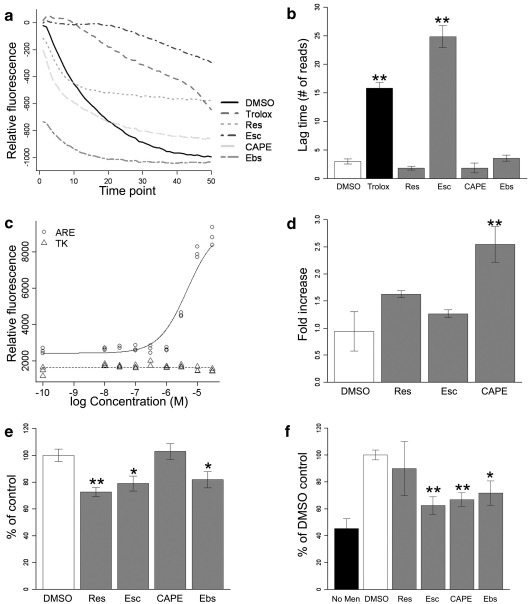
Cellular pathways activated by best-hit molecules. (a) Typical traces from TRAP assay. There was a 1-min delay between each reading of the plate. (b) Summary of TRAP assay results, showing that of the best-hit molecules tested, only esculetin significantly delayed decay of R-PE (*p* = 0.0051 for both esculetin and Trolox, Wilcoxon test). (c) Typical dose–response curves for the effect of CAPE on induction of 4 × ARE-TK-EGFP (solid line) and TK-EGFP (broken line). (d) Summary of the induction of ARE-TK-EGFP. Only CAPE significantly induced ARE-mediated gene transcription (*p* = 0.0022; Student's *t* test). (e) 5-LOX activity assay. Resveratrol, esculetin, and ebselen all significantly reduced LTB_4_ synthesis by 5-LOX (*p* = 0.0028, 0.014, and 0.028, respectively; Student's *t* test). (f) NF-κB activation assay. Menadione (10 μM) induced translocation of NF-κB to the nucleus (white bar). This translocation could be significantly inhibited by CAPE, esculetin, and ebselen (*p* = 0.00021, 0.0013, and 0.031, respectively; Wilcoxon test). All results shown are means ± SEM. All pairwise tests were performed only after statistical significance was discovered with the appropriate combined test (ANOVA or Kruskal–Wallis) and were adjusted for multiple comparisons.

To determine the ability of each molecule to scavenge free radicals, the TRAP was measured relative to 6-hydroxy-2,5,7,8-tetramethylchroman-2-carboxylic acid (Trolox; a water-soluble analogue of vitamin E) [35]. R-PE fluorescence decays in the presence of free radicals produced by controlled decomposition of AAPH. Free radical scavengers can buffer the radicals produced from the decomposition of AAPH and delay the decay of R-PE. The length of the lag time before R-PE decay begins is therefore proportional to the free radical scavenging ability. To control for the bleaching of R-PE fluorescence by repeated excitation, relative fluorescence was calculated by subtracting the R-PE fluorescence in the absence of AAPH. Each of the best-hit molecules and ebselen were tested in this assay, along with Trolox as a positive control. Typical traces from the TRAP assay are shown in Fig. 7a. Of the molecules tested, only esculetin significantly delayed decay of R-PE, suggesting that only esculetin can directly buffer free radicals (*p* = 0.0051, Wilcoxon test, Fig. 7b). The other hit molecules must therefore be acting through cellular antioxidant pathways.

#### Induction of antioxidant response element

We have previously shown that NSC34 cells expressing G93A-SOD1 have down-regulated a number of “programmed cell life” genes, the expression of which is regulated by the transcription factor Nrf2 (nuclear factor erythroid 2-related factor 2) acting through the ARE [52]. Using a GFP reporter assay, we found that ebselen, the positive control in the library screen, can induce transcription of genes under the control of the ARE [37]. We therefore used this assay system to determine whether any of the best-hit molecules identified in the library screen act through the ARE pathway. Only CAPE induced transcription of GFP under the control of the ARE, with an EC_50_ of 9.5 μM (*p* = 0.0022; Student's *t* test, Figs. 7c and d). CAPE did not increase GFP fluorescence in the control TK-ARE-expressing cells, showing that increased GFP production required the ARE. Resveratrol and esculetin did not increase GFP fluorescence.

#### Inhibition of 5-lipoxygenase

There is increasing evidence for the role of the proinflammatory eicosanoid LTB_4_ in G93A-SOD1-mediated ALS, with increased levels reported in G93A-SOD1 mouse spinal cord [53] and cultured astrocytes [54]. Given that LTB_4_ can increase reactive oxygen species production [55] and NDGA, an inhibitor of 5-lipoxygenase (5-LOX)-mediated LTB_4_ production, reduced oxidative stress in NSC34 cells as well as increasing survival of G93A-SOD1 mice [53], we investigated whether any of the best-hit molecules were affecting 5-LOX activity. NSC34 cells were treated with 10 μM arachidonic acid in the presence and absence of 10 μM compound for 3 h before the culture medium was harvested and analyzed for LTB_4_. Resveratrol, esculetin, and ebselen were all found to significantly inhibit LTB_4_ production from arachidonic acid (*p* = 0.0028, 0.014, and 0.024, respectively; Student's *t* test), whereas CAPE had no effect on LTB_4_ levels (Fig. 7e). This result contrasts with a report describing CAPE as a 5-LOX inhibitor in mouse peritoneal macrophages [56].

#### Inhibition of NF-κB

Oxidative stress can activate the transcription factor NF-κB, leading to further cellular stress, and CAPE has previously been described as an inhibitor of NF-κB activation [49]. To investigate whether CAPE and/or any of the other best-hit molecules acts through inhibition of NF-κB, NSC34 cells were pretreated with each compound for 1 h before oxidative stress was induced by 10 μM menadione. NF-κB translocation was increased by over 2.5-fold by menadione (*p* = 0.00021) and was partly inhibited by CAPE, esculetin, and ebselen (*p* = 0.00021, 0.0013, and 0.031, respectively; Wilcoxon test, Fig. 7f).

## Discussion

We have used an in vitro screening cascade to assay the Spectrum Collection of 2000 compounds and several known candidate antioxidant compounds for their ability to reduce serum withdrawal-induced oxidative stress in a motor neuron cell line. The hits have been further refined in subsequent assays of oxidative stress and viability in motor neuron cells expressing an ALS-associated SOD1 mutation (G93A), and compounds likely to be able to access the CNS were selected based on their predicted biochemical properties. The most attractive compounds, resveratrol, esculetin, and CAPE, were then tested for their ability to rescue primary MNs from trophic factor withdrawal-induced oxidative stress and cell death. Although these molecules were all capable of reducing oxidative stress, they seemed to be acting via different mechanisms.

The Spectrum Collection consists of the NINDS Custom Collection, a selection of pure natural products and a small number of endocrine disruptors and toxic substances. The NINDS collection includes 640 drugs approved by the Food and Drug Administration, drugs that have reached late-phase human trials, and drugs likely to affect CNS function. Such a collection provides both structural and biological diversity for screening programs. Any hits in approved drugs should have a rapid route to the clinic, because they are known to be drug-like and safe in humans, whereas natural products are a potentially rich source of antioxidants and have provided the starting points for many approved drugs throughout the history of drug development.

Although many of the hits from the initial library screen have previously been described as antioxidants, other molecules not previously recognized as having antioxidant activity were also identified. Perhaps of more interest, some well-characterized antioxidants present within the library, including glutathione and vitamins C (ascorbic acid) and E (α-tocopherol), were not identified as hits. This may be because they failed to reach the site of oxidative stress at concentrations sufficient to have an effect. The ability of glutathione to cross plasma membranes is limited, which may account for its poor performance in the library screen. However, OTCA, a cell-permeative precursor of glutathione [57], tested as one of the known antioxidant molecules, also failed to rescue NSC34 cells from serum withdrawal-induced oxidative stress, although whether OTCA treatment leads to increased glutathione levels in NSC34 cells was not determined (Table 1). Similarly, vitamin E is a potent antioxidant, but it is highly lipid-soluble and therefore tends to partition into lipid-rich compartments, such as the plasma membrane, where it may be unable to buffer cytosolic free radicals. In support of this, the water-soluble vitamin E analogue Trolox was capable of reducing cytosolic oxidative stress. Although vitamin C has antioxidant activity, many previous in vitro studies have found it to be unable to reduce oxidative stress [58,59] or used much higher doses than the 10 μM used in the Spectrum library screen [60–62]. Alternatively, the failure of such free radical buffers may indicate that direct buffering of free radicals is not sufficient to rescue the toxic gain of function caused by mutant SOD1 in ALS. This is further supported by considering that of the best-hit molecules, only esculetin was capable of directly buffering free radicals. Esculetin is one of at least 12 coumarins or coumarin derivatives in the Spectrum library, but was the only one to be identified as a hit in the initial screen, even though fraxetin, another coumarin present within the library, has been shown to be more potent at scavenging superoxide [63]. This suggests that the protective effects of esculetin observed may be mediated via cellular signaling pathways and again supports the hypothesis that free radical buffering may be insufficient to rescue oxidatively stressed NSC34 cells.

The best-performing molecules identified from the Spectrum library screen, resveratrol, esculetin, and CAPE, have all previously been identified as naturally occurring antioxidants [45–48,64]. Resveratrol is found in red wine, esculetin is present in tobacco leaves, and CAPE is an active component of the bee product propolis. In NSC34 cells, each of these molecules was capable of activating antioxidant cellular pathways (Fig. 7). Resveratrol initially seemed to be an attractive compound, with extensive in vitro and in vivo studies reviewed elsewhere [45]. However, the subsequent assays showed resveratrol to be less effective than either esculetin or CAPE, and a review of the literature showed that although the in vitro studies have mostly been successful, they often require high doses of resveratrol and in vivo studies have shown rapid metabolism and failed to detect resveratrol in the CNS [65]. Taken together, this suggested that resveratrol is unlikely to be effective in vivo.

Esculetin and CAPE performed comparably in the in vitro screening, and both activated a variety of, albeit different, cellular pathways. Both esculetin and CAPE inhibited NF-κB activation, and esculetin also inhibited 5-LOX, representing two pathways that when activated lead to inflammation [66,67]. CAPE was also able to activate the Nrf2–ARE pathway (Figs. 7c and d), a pathway we have previously shown to be down-regulated in NSC34 cells expressing mutant SOD1 and MNs isolated from cases of familial SOD1-associated ALS [52]. The structure of CAPE, including a hydrophobic/aromatic moiety with a hydroxyl group, is consistent with known Nrf2 activators, which are thought to react with Keap1 (Kelch ECH-associating protein 1; the cytoplasmic Nrf2 regulator), thereby releasing Nrf2 for translocation to the nucleus [68]. Genes up-regulated by Nrf2 include the antioxidants glutathione peroxidase, glutathione reductase, and heme oxygenase 1; enzymes involved in glutathione synthesis; and NADPH-regenerating enzymes [69,70].

There are multiple reports showing beneficial effects of both esculetin [47] and CAPE [50,71–73] in vivo, although only CAPE has shown positive effects in the CNS. There is evidence to suggest that esculetin is efficiently removed by the liver [74] and therefore may not achieve levels sufficient to be effective in the CNS. CAPE has been shown to be well absorbed into plasma after oral administration [75], and although it was found to have a short half-life in rat plasma in vitro, it was metabolized to caffeic acid [76], which was also effective in serum-withdrawn NSC34 cells and showed a positive trend when tested in G93A-SOD1-expressing cells. Other caffeic acid derivatives were also identified in the initial library screen, including chlorogenic acid and rosmarinic acid (effective in NSC34 cells, but failed to reach statistical significance in G93A-SOD1-expressing cells), suggesting that metabolites of CAPE may still be effective in vivo. Taken together with the finding that the half-life of CAPE in human plasma in vitro was more sustained than in rat plasma [76], this makes CAPE an attractive candidate to take forward for in vivo studies. We are now currently investigating the pharmacokinetic profile of the best-hit molecules in mice, with the intention of progressing to studies of disease-modifying effects in the murine G93A-SOD1 transgenic model of ALS.

## Figures and Tables

**Table 1:** Known antioxidants tested in NSC34 oxidative stress and viability assays

Molecule	Reported antioxidant activity	EC_50_ (μM)	Max % DCF decrease	Protective in G93A cells?		ALS-specific findings
				Basal DCF	MTT	
*N*-(4-hydroxyphenyl)arachidonoyl ethanolamide (AM404)	Anandamide transport inhibitor, biochemical antioxidant [77]	4	80	Yes	Yes	Not found
Apocynin	Inhibits NADPH oxidase	—	—	ND	ND	Increased SOD1^G93A^ mouse survival [4]
Flupirtine	Decreases mitochondrial free radical generation [78]	7	67	No	ND	Not found
α-Lipoic acid	Activates Nrf2 [79]; scavenges ROS	—	—	ND	ND	Increased SOD1^G93A^ mouse survival [80]
Nordihydroguaiaretic acid	5-Lipoxygenase antagonist [53]	4	108	Yes	Yes	Increased SOD1^G93A^ mouse survival [53]
2-Oxo-l-thiazolidine-4-carboxylic acid (OTCA)	Cell-permeative glutathione precursor [57]	—	—	ND	ND	Not found
4-Hydroxy-2,2,6,6-tetramethylpiperidine 1-oxyl (Tempol)	Free radical scavenger [81]	—	—	ND	ND	Not found
Trolox ((±)-6-hydroxy-2,5,7,8-tetramethylchromane-2-carboxylic acid)	Water-soluble vitamin E analogue (ROS scavenger) [82]	1	79	Yes	No	Increased viability of SOD1^G93A^-expressing motor neuron cell line [82]

Molecules were considered to be protective in G93A-NSC34 cells if they significantly reduced basal oxidative stress or increased viability after 10 μM menadione treatment (by MTT) at concentrations up to 10 μM (*p* < 0.05, ANOVA). ND, not determined.

**Table 2:** Summary of best-hit molecules from screen in NSC34 cells and their in silico properties

Molecule	Serum-withdrawn NSC34		G93A cells		MW	Log*P*	H donor	H acceptor	Rule of 5	PSA (Å^2^)
	EC_50_ (μM)	Max % DCF decrease	DCF (% of basal)	MTT (% of control)						
Resveratrol	4	54	72	134	228.3	3.09	3	3	Pass	60.68
Esculetin	1	80	−7	193	178.1	1.42	2	4	Pass	66.76
Caffeic acid phenethyl ester	2	94	36	254	284.3	3.57	2	4	Pass	66.76
Quercetin	5	43	15	160	302.2	1.63	5	7	Pass	127.45
Morin	14	123	33	128	302.2	1.63	5	7	Pass	127.45
Dipyrone	1	75	61	122	333.3	0.38	1	7	Pass	150.36
Epicatechin pentaacetate	5	95	62	159	500.5	2.44	0	11	Fail	140.72
Chlorogenic acid	0.6	50	67	134	354.3	-0.34	6	9	Fail	164.75
Quercitrin	0.9	43	74	124	448.4	0.59	7	11	Fail	186.37

Rule of 5 and molecular polar surface area calculations were performed using Pipeline Pilot software. MW, molecular weight; Log*P,* octanol/water partition coefficient; H donor, number of hydrogen bond donors; H acceptor, number of hydrogen bond acceptors; PSA, polar surface area (Å^2^).
